# Horizon scanning from panel discussions at the EUGOGO Global TED Forum 2025 London

**DOI:** 10.1038/s41433-025-04197-z

**Published:** 2026-01-07

**Authors:** Vickie Lee, Mario Salvi, Anja Eckstein, Colin Dayan, Karim Meeran

**Affiliations:** 1 Imperial College London, London, UK; 2 Fondazione IRCCS Cà Granda Ospedale Maggiore Policlinico Milano, Milano, Italy; 3 University Duisburg-Essen, Essen, Germany; 4 University of Birmingham, Birmingham, UK

**Keywords:** Thyroid diseases, Conferences and meetings

## Abstract

This is a summary of some of the panel and round table discussions held under the auspices of the European Group on Graves’ Orbitopathy (EUGOGO) GlobalTED Forum at Imperial College London in September 2025 to identify unmet needs, explore future directions, and propose practical strategies for improving the management of Thyroid Eye Disease (TED).

## Measuring what matters: refining assessment tools

Current TED assessment tools remain limited by subjectivity and lack of standardisation. Harmonisation of imaging protocols across centres was identified as a critical step for reproducibility. Artificial intelligence (AI) offers opportunities for objective image analysis, relapse prediction, and personalised treatment planning, but reliable use requires integration of imaging with reliable clinical data. Federated learning models were endorsed to support multicentre collaboration while maintaining data privacy. Combining inflammatory scales, biomarker panels, and imaging thresholds should improve disease activity assessment. Patient self-monitoring tools and adaptive educational platforms could enhance engagement and longitudinal data collection.

## Defining meaningful outcomes: diplopia, appearance, and quality of life

Diplopia was identified as the most important patient-centred outcome due to its profound functional impact. The commonly used Gorman score lacks objectivity; instead, measures such as Goldman’s field of binocular single vision, ductions in multiple gaze positions, and orthoptic assessment of squint angles including cyclotorsion were favoured. Weighted scoring systems prioritising central and downgaze alignment may better reflect function. Clinically meaningful endpoints include reduced prism use, improved binocular function, and avoidance of major rehabilitative surgery. Emerging virtual-reality-based assessments could enable automated, standardised measurement.

Appearance was the second major outcome domain, with even mild changes significantly affecting psychosocial wellbeing. AI-based 3D reconstruction may in future quantify symmetry changes. Future quality-of-life measures should aim to capture patient-reported outcomes to reflect lived experience.

## Integrated treatment of thyroid and ocular disease

Mechanism-based therapy should aim to minimise systemic immunologic burden. Direct blockage of the TSH receptor (TSH-R) is possibly the most effective direct therapy but remains hypothetical at this stage. FcRn inhibitors may reduce IgG thyroid-stimulating immunoglobulins (TSI) levels but may require monitoring for hypoalbuminaemia and dyslipidaemia. FcRn activation after therapy discontinuation can transiently elevate immunoglobulin levels, complicating antibody normalisation. Addressing the pathologic immune reaction may induce the long-term remission eg belimumab and rituximab (RTX), which targets B cells producing TSI improved remission rates of hyperthyroidism in juvenile Graves hyperthyroidism and Tocilizumab (TCZ) which targets IL6 have been shown efficacy in TSI level and inflammation reduction.

## Relapse/Reactivation/Flares

This remain a key challenge and are most commonly observed after steroid and IGF-1R inhibitor treatment and appear to be less frequent after TCZ or RTX treatment. Evidence on retreatment strategies is limited. Fixed-dose regimens may not accommodate the clinical heterogeneity and duration of the progressive phase of the disease. Future trials should target high-risk populations and standardise definitions of relapse, remission, and response. Early combination treatment, e.g. IGF1 with FcRn inhibitors/biologicals warrants further investigation in larger cohorts and longer follow up. Long-term efficacy data are sparse and better understanding of the autoimmune trajectory is needed to identify high risk patients.

## Hearing loss associated with IGFR-1 antagonists

Permanent sensorineural hearing loss is relatively uncommon but can be clinically significant. This can often be unilateral, affecting high frequencies with older patients at higher risk. There is no evidence for prophylaxis, but urgent audiometry and discontinuation of treatment are recommended. There is no proven effective treatment for the induced hearing loss. Intratympanic steroids, hearing aids, or cochlear implants may be considered in refractory cases. Topical IGF-1 therapies remain experimental.Collaboration with ENT specialists is essential, particularly in severe or high-risk cases.

## Surgical timing after teprotumumab

Optimal timing of rehabilitative surgery following teprotumumab remains debated. Expert consensus suggests a 6–12-month interval, with 1 year  considered  safer. Earlier orbital decompression may be justified for vision-threatening proptosis but may carry increased relapse risk.

## Orbital radiotherapy (ORT)

Some panellists favour ORT as an effective, low-relapse and DON rate reducing, immunomodulatory therapy in TED. Colleagues from the USA indicate that ORT can be safely administered after teprotumumab.

## Biomarkers

Biomarkers hold potential across diagnostic, predictive, and pharmacodynamic domains. TSI/TRAb are the most potent biomarker available so fare. Higher titres identify patients at risk for relapse and more severe course of TED. Dynamic changes in titre, affinity, and rate of rise may provide more insight than absolute values. The impact of rising/high TSI titres on surgical timing remains controversial, reflecting divided expert opinion.

## Empowering patient groups

Limited patient access to clinical trials was highlighted. A centralised, regularly updated EUGOGO clinical trial register was proposed to improve visibility and recruitment. Collaboration with patient advocacy groups and a stronger social media presence could increase awareness and engagement. Educational initiatives, including multilingual webinars, were recommended to empower patients, improve research participation, and enhance clinical trial retention.

## Addressing global disparities

Access to novel biological treatments remains inconsistent and cost-prohibitive in many regions. Some countries, such as China, have introduced lower cost IGFR1 biosimilars. Where biologics are unavailable, evidence-based use of steroids, and other non-specific immunosuppresants, radiotherapy, and surgery can still achieve reasonable outcomes. Guidelines should remain flexible and locally relevant, recognising validated non-biologic pathways. The question remains whether investment in surgical training and multidisciplinary centres may yield greater population benefit than limited access to high-cost agents.

Ethnic and anatomical variations should inform care, as ‘white-eye’ TED phenotypes being common in non-white patients. Addressing psychosocial impact through mental health support and management of dry eye disease can substantially improve quality of life.

## Prevention: the missing piece

Prevention and early recognition remain critical. Smoking cessation remains the most effective intervention, and maintaining euthyroidism through optimal management of hyperthyroidism is essential. Selenium may help in deficient populations, although evidence is mixed. Early treatment with statins in patients with hypercholesterinaemia may reduce incidence and severity of TED and improve treatment outcomes. Research is needed as to whether optimising vitamin D, endocrine disruptors and air pollution, stress management, and Mediterranean dietary patterns offer additional protection. Vigilance recommended for perioperative stressors, including cataract or strabismus and orbital decompression surgery. Primary immunomodulatory treatment at early onset of autoimmune hyperthyroidism needs to be explored.

## Conclusion

The discussions emphasised the need for an integrated, standardised, and patient-centred framework for TED management. Progress will depend on harmonised assessment tools, validated biomarkers, AI-enabled analytics, and equitable access to effective therapies. Empowered patients, multidisciplinary collaboration, and preventive strategies remain central to improving global outcomes.

*With Thanks to the Panellists & Moderators: Tomasz Bednarczuk, Jason Brant, Kelvin Chong, Chrysoula Dosiou, Anja Eckstein, Claire Feeney, Nicole Fichter, Don Kikkawa, Michael Kazim, Andrea Kossler, Onur Konuk, Vickie Lee, Karim Meeran, Josian Phillips, Mario Salvi, Marius Stan, Gangadhara Sundar, and Milos Zarkovic*.

